# An innovative electronic health records system for rare and complex diseases

**DOI:** 10.1186/1471-2105-16-S19-S4

**Published:** 2015-12-16

**Authors:** Alessandra C Faria-Campos, Lucas A Hanke, Paulo HS Batista, Vinicius Garcia, Sérgio VA Campos

**Affiliations:** 1 Department of Computer Science, Universidade Federal de Minas Gerais, Antônio Carlos Avenue, 6627, Pampulha, Belo Horizonte, MG, 30123-970, BR; 2 INMETRO, Nsa.Sra.das Graças Avenue, 50, Xérem-Duque de Caxias, RJ, 25250-020, BR

**Keywords:** Electronic Health Record, Laboratory Information Management Systems, Workflow

## Abstract

**Background:**

There exists a large number of rare and complex diseases that are neglected due to the difficulty in diagnosis and treatment. Being rare, they normally do not justify the costs of developing an especialized Electronic Health Record (EHR) system to assist doctors and patients of these diseases. In this work we propose the use of Computer applications known as Laboratory Information Management Systems (LIMS) to address this issue.

**Results:**

In this work we describe a fully customizable EHR system that uses a workflow based LIMS with an easy to adapt interface for data collection and retrieval. This system can easily be customized to manage different types of medical data. The customization for a new disease can be done in a few hours with the help of a specialist.

**Conclusion:**

We have used the proposed system to manage data from patients of three complex diseases: neuromyelitis optica, paracoccidioidomycosis and adrenoleukodistrofy. These diseases have very different symptoms, exams, diagnostics and treatments, but the FluxMED system is able to manage these data in a highly specialized manner without any modifications to its code.

## Introduction

There is a large number of rare and complex diseases that are not well studied. Being rare they are often neglected because they do not affect a large number of patients. However, for the patients afflicted by them treatments are essential. Besides, while each disease is rare, when accounted for together, they represent a serious health problem, potentially incapacitating a large number of people, and increasing health related expenses significantly.

For most of these diseases diagnosis and treatment depends on an expert doctor, often unavailable. The use of Electronic Health Record (EHR) systems to store and analyze data from these diseases can help in making diagnosis and treatments more effective. In this work we propose the use of a LIMS, or Laboratory Information Management System to develop an EHR that is simple to customize for complex diseases and that can be easily changed to adapt to new diseases, making it possible to apply this technology for neglected diseases.

LIMS are computational tools developed to integrate and manage laboratory data that give emphasis to quality assurance and aim to generate results in a consistent and reliable way [1]. Several LIMS are available nowadays as academic, proprietary and open source applications. Some examples include SQL LIMS [2], LabSoft [3], LabWare [4] (proprietary applications), FreeLIMS [5] an open source application developed by Labmatica and the academic systems developed by Hendrick [6], Quo [7], Tharayil [8] and Sanchez [9]. Melo [10] has proposed a new system - SIGLa - a workflow based LIMS designed to allow it to adapt its activities and processes to various types of laboratories. A workflow can be defined as a sequence of steps and tasks executed according to a set of rules and procedures in order to complete a process. A workflow can be a sequential progression of activities, or a complex set of processes occurring concurrently and eventually impacting in others, according to a set of rules [11]. Other LIMS incorporate this concept, such as the systems developed by Wilkins [12] and Esterling [13] that manage data from proteomics laboratories.

The need for specific LIMS is particularly important for medical laboratories and facilities since for medical applications, existing systems are frequently focused on maintaining the doctors schedule and keeping general annotations on the patients conditions. They are usually generic in the sense that they are not customized for specific illnesses, or conditions that require complex analyses. As a consequence, the data stored cannot be used for a detailed analysis and cannot be easily integrated with other systems.

Existing systems tend to fall in one of two categories. They can be too rigid in the types of data that can be stored, limiting severely the symptoms, exams, diagnostics that can be used, allowing using and editing data only from the limited set of pre-specified information that has been coded into the system. Frequently doctors complain that such systems force them to enter the data in ways that are not appropriate because there is no way of changing the types of data that are accepted.

As a way of compensating for this problem, the other type of system is too generic, allowing the doctor to enter free text describing the patients consultation. Data entered in this way is very difficult to analyze, because each doctor specifies a different set of symptoms, treatments and so on. Frequently data from one consultation to the other is not comparable [14,15].

In this work we propose a different approach, making it possible to standardize the types of data entered by defining them in a workflow. These can be changed easily, incorporating new knowledge without changes to the system. It can be used in very flexible ways, for example, if different doctors follow different diagnostic strategies, that is, ask different questions, and request different exams, the workflow can incorporate both methods, and let the doctor choose which one to use.

Data entered in this way is structured to make it easy to analyze it later. Data is not entered in free text format, but in formats that have fixed types and requirements, which simplifies posterior analysis.

This system, called FluxMED, can be used also to connect to external equipments and analysis software. Data from clinical analyzers can be directly imported in FluxMED, and connections to other systems are available. We have implemented connections to the R statistical package and to the Weka data analysis system. Other connections can be easily implemented.

We have used FluxMED to develop EHR systems for three different diseases that are complex, difficult to diagnose and to treat. But because they are not common diseases, EHR systems aimed at them are non existant or very difficult to access. FluxMED has been able to model data from patients of neuromyelitis optica, paracoccidioidomycosis and adrenoleukodistrofy and enable doctors to use the system to initiate and follow patient treatments. Data from these datasets will later be used on data analysis systems to identify patterns and conditions that can help treating the patients and improving their life.

An important aspect of the FluxMED system is that creating a workflow for a new disease takes only a few hours with the help of a specialist. There is no need to change the system in any way. With some training the doctors can themselves specify the workflow and create a new EHR system. Moreover, new systems can be integrated with existing ones, so one EHR system can serve several specialities, making it simpler to maintain the data, train users and extend the system. FluxMED allows users to compare data between different but related diseases in search of common aspects that can be considered for a treatment, but which would have been very difficult to identify if no integrated system was available.

FluxMED is based on the SIGLa system [10], managing data specified in workflows. It has an easy to use Web interface, making it possible to access data from any computer without previously installing a viewer software. It has several security features that prevent unauthorized access, including different permission sets for users to access different types of data depending on their authorization. The system can be accessed at the address below with login/password: guest/gu3st. All data in the web site and in this paper is fictitious and do not represent real patients.

http://syrah.luar.dcc.ufmg.br/FluxMED

## FluxMed development

The FluxMED system has been constructed using Java and uses MySQL as database server and Apache Tomcat as Web server. The system has a web interface and runs in the main existing Web browsers. Workflow files are uploaded in the system through the interface and the activities created in the workflow construction are interpreted as links by the system. The workflows have been constructed in the XPDL format using the application Together Workflow Editor (TWE) [16]. In FluxMED the EHR systems are defined as sequences of activities. An activity represents events such as a consultation, an exam or test performed, or a diagnosis determined. Specific information, such as names, dates, values for specific exams or doctor analysis are represented as attributes in the workflow definition. Therefore, the user can define the characteristics of the attributes of each activity, such as its types, the range of values that each attribute can assume, its formats or even define auto-calculated attributes generated from other attributes.

Several types of attributes contribute to the generality of the FluxMED implemented EHRs. Traditional types of attributes are supported, such as integer or real numbers, which can have specified ranges, or strings containing text. Other types include the register attribute, which ensures that the values entered are already present in a separate table. For example, the name of the doctor may be specified to contain only values that have been already registered in the system, avoiding problems with misspelling names. Other types of attributes can be chosen from a previously specified list of values. One type of attribute allows the user to enter an unlimited number of values, which themselves can consist of a set of other attributes. This feature is useful, for example, when the patient relates a series of symptoms, each with an associated date or value. The system also allows each attribute to have an example and a hint field, making it easier to use the system. All of these types of attributes and their functionalities can be specified in the workflow, and can be added or modified by the user without changing system code.

**Figure 1:**
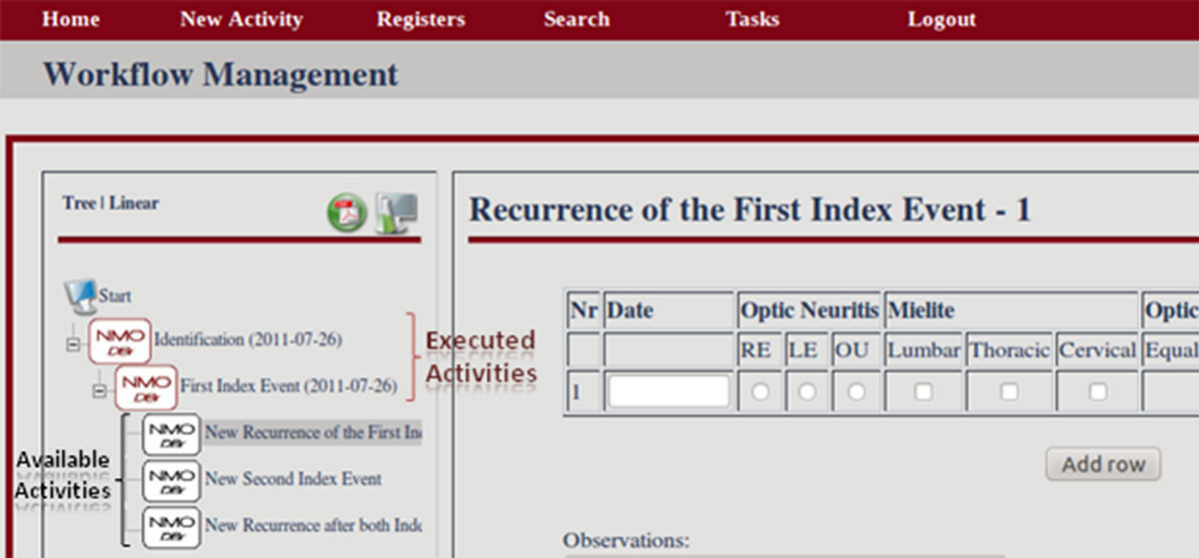
**FluxMed web interface -- Electronic forms for the NMO EHR system with executed activities shown in red and activities available for execution shown in black**. http://syrah.luar.dcc.ufmg.br/FluxMED -- guest/gu3st

The FluxMED System has an easy to use interface where activities that have already been executed are represented by icons in a different color from those for activities available to be executed (Figure 1 shows part of the screen). The system guides the user through the entire process, informing which activities are available for execution. Adittionally, this execution can also be done automatically through the upload of a file containing the attributes values. The workflow is entirely modeled and built on TWE and therefore the workflow contains all the information regarding the entire process. As a consequence, to change the type of data being stored, only the workflow has to be modified.

## EHR systems developed

To illustrate the usefulness of FluxMED, we have modelled and implemented EHR systems for three diseases: neuromyelitis optica (NMO), paracoccidioidomycosis(PCM) and adrenoleukodistrofy (ALD). All systems have been specified by a specialist in the disease, and the workflow development has been done by a informatics student working with the specialist. Tipically development takes a few hours spread over a number of sessions which interactively refine the workflow until the system is considered finished. All systems have been tested by doctors which verified that the system can be used for real patient consultation. Two systems, NMO and ALD have not yet been deployed in actual consultations. The PCM system has been used already to store data from several patients, and doctors are now completing the database with previous consultation data to allow the continuation of the treatment in the system and a complete analysis of existing patient data.

### Neuromyelitis Optica -- NMO

Neuromyelitis optica(NMO) is an important central nervous system disease. NMO is an idiopathic inflammatory demyelinating disease of the central nervous system (CNS) most frequently characterized by recurring attacks of optic neuritis and myelitis. It can be distinguished from conventional multiple sclerosis on demographic, clinical, neuroimaging, cerebrospinal fluid and serological grounds [17]. NMO spectrum disorders are highly prevalent among the demyelinating diseases of the CNS in Southeastern Brazil and a significant amount of data from NMO patients is generated from medical care. The collection and analysis of these data will result in a better characterization of the disease and therefore, there is a strong need for computational tools and databases to collect, store, manage and retrieve NMO data in order to help improve research and medical care. This work has been done in cooperation with Dr. Marco Aurélio Lana, from the Center for Investigation of Multiple Sclerosis (CIEM) at Federal University of Minas Gerais (UFMG).

**Figure 2:**
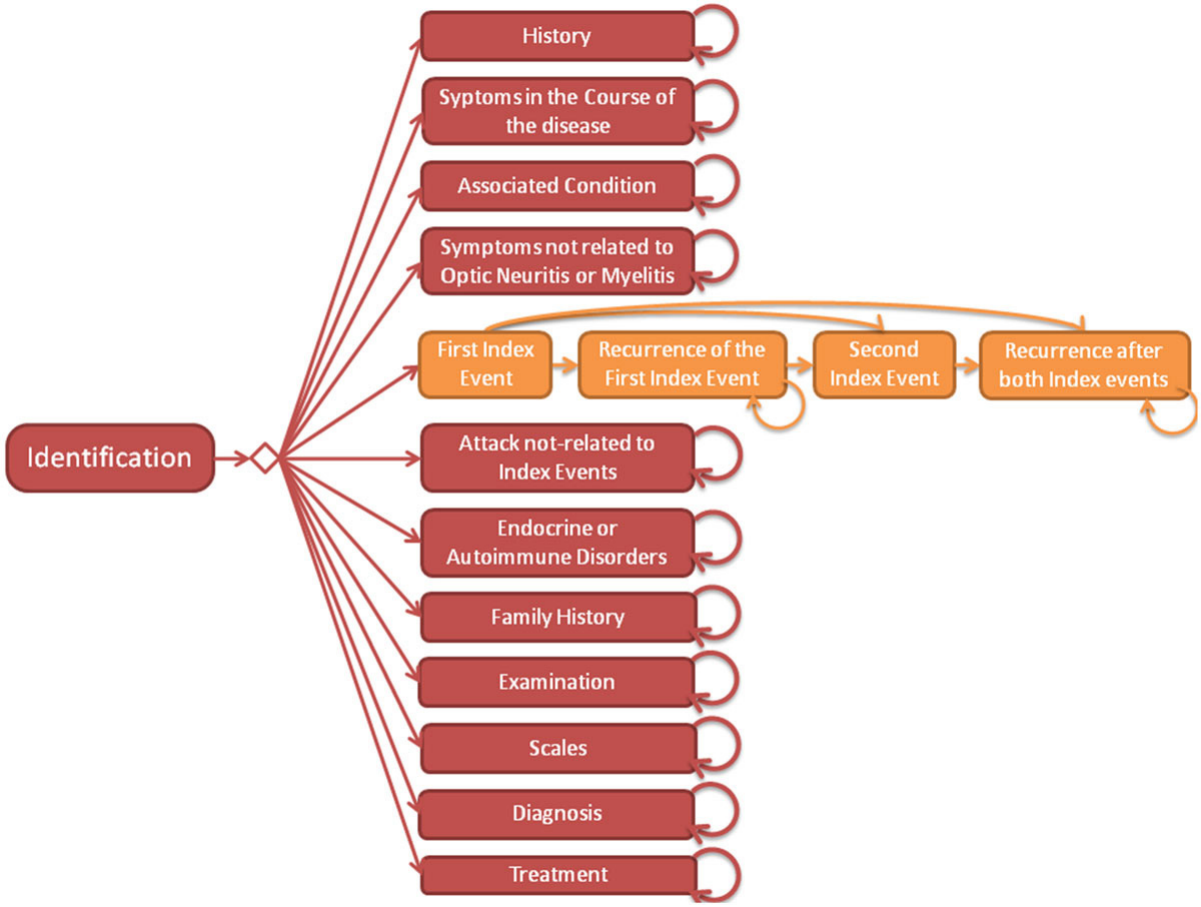
**The Neuromyelitis Optica (NMO) workflow definition**.

A complete workflow for storage and management of data from NMO patients has been created to be used in the FluxMED System, along with a database for data storage [18]. The workflow consists of 16 activities (Figure 2) and each of those has an individual screen in the system. The first activity is the *Identification *which requires general information from the patient, such as name, age and level of education. In this activity, a numeric identifier is automatically generated for the patient and this id is used as a tag to follow all data regarding the patient in the database. Other paciente related informations like *City*, *State *and *Country *are collected along this unique pacient identifier. *The Date of First Evaluation *is an important information which will be used along with other dates through the process by the doctor in the disease diagnosis.

Each activity has its own set of attributes and the formats for the data are defined during the workflow definition. These activities represent all the steps used in medical evaluation of NMO patients and allow the user to store information on personal data, symptoms and exams to which the patient has been submitted. For some activities, the sets of attributes are represented by tables with flexible size. The activity *Family History *is an example of such an activity where the user can add an adittional row for each pacient's family member.

### Paracoccidioidomycosis -- PCM

Paracoccidioidomicosis(PCM) is a typical Brazilian disease, caused by the yeast *Paracoccidioides brasiliensis*. The most common form of infection is through inhalation of spores. The spores cause infection of the lung epithelium and can spread to other organs. The disease mainly affects farm workers who are exposed to contaminated soil during labor [19]. This disease represents an important Public Health issue, due to its high incapacitating potential and the amount of premature deaths it causes if untreated. The analysis and management of PCM related data presents several challenges. One of the challenges is related to data acquisition during patient evaluation and diagnosis. The Center of Training and Reference on Infeccious-Parasitary Diseases at UFMG (CTR-DIP-UFMG), coordinated by Dr. Enio Pietra, has developed a protocol for clinical analysis of PCM patients. This protocol includes a large number of clinical variables that are assessed in each medical examination, including x-ray and serology tests, which are also used in tracking the disease progression.

**Figure 3:**
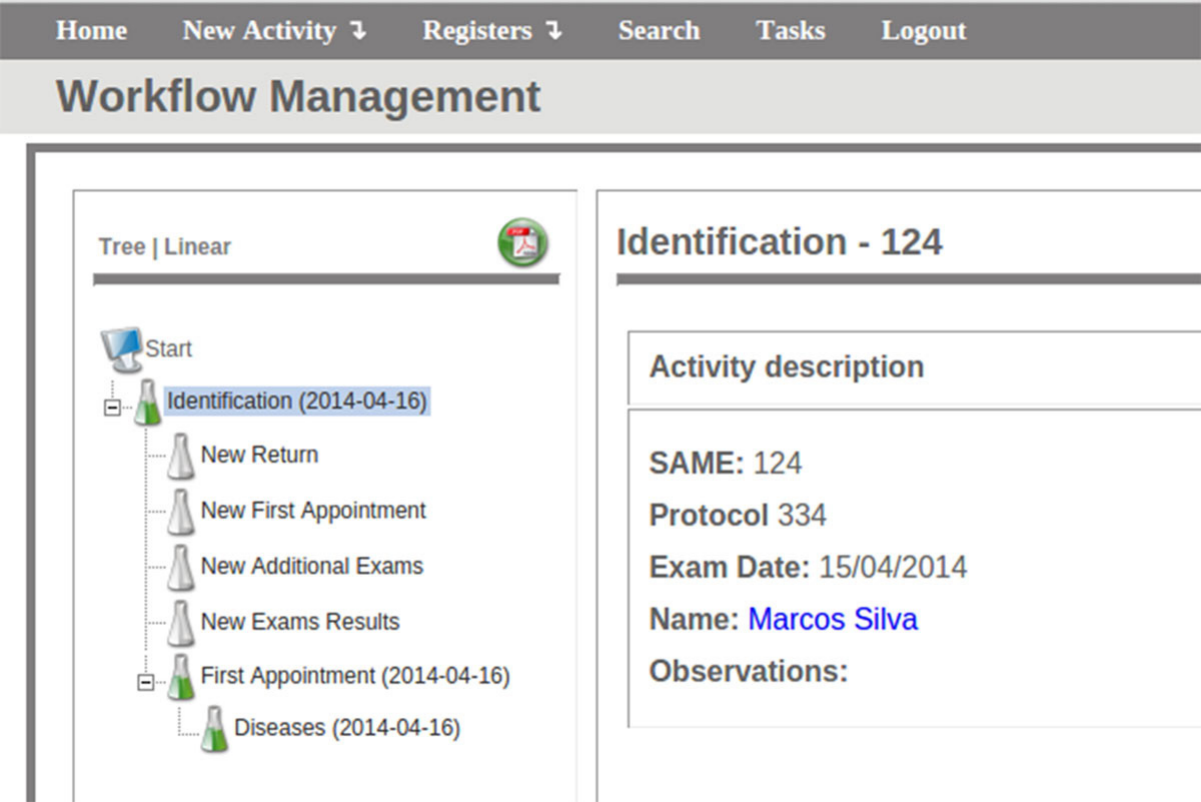
**FluxMed web interface -- Electronic forms for the PCM EHR system**. http://syrah.luar.dcc.ufmg.br/FluxMED -- guest/gu3st

We have modelled this protocol in a workflow and used it to create an EHR system in FluxMED. This system is currently in use at UFMG, where it is helping manage patient's data and assisting in defining treatment duration and other conditions (Figure 3) [20].

The first step in FluxMed PCM, as well in others systems is the login. Access on the system is only granted to valid users. Pacient identification is the first activity in the workflow. FluxMed PCM requires less data than NMO about the pacient medical records and shows fields about the main pacient information, like protocol and the SAME, an unique number that identifies the pacient Personal Medical History in the hospital database. Also, a set of other patient information is stored as well.

After all the information is filled in the system is possible to add information about the first consultation of the patient, return visits, exams and exam results. The first consultation generates data which are stored and automatically related to the patient without the risk of losing or mixing, as in the storage of paper. Current patient symptoms are stored, as well the major complaints and 35 possible medical conditions. These medical conditions are showed in an list of radio buttons with options to indicate the presence or absence of each one. Under the *First Consultation *activity its possible register a set of other informations, like patient disease history, covering 29 present and previous diseases. There are special fields to allow the inclusion of information on diseases that have not been covered by the system. Due to FluxMED PCM architecture these additional diseases can be added to the radio button list without modifications on the code or data loss, simply adding them to the workflow.

FluxMED also covers the data obtained by exams in the respiratory, cardiovascular and digestive systems, storing possible alterations which can help in the diagnosis. All this data can be registered in the system as many times as the patient makes a consultation. At the end of the data collection process the system shows fields to store diagnostic hypothesis, exams and drug prescriptions.

### Adrenoleukodistrofy -- ALD

The Inborne Errors of Metabolism Laboratory at UFMG works with patients from the public health system in Brazil, identifying and treating rare genetic diseases that are difficult to diagnose but often have treatments available if diagnosed early. In the last 50 years, more than 500 genetic diseases that involve metabolic processes have been identified. These diseases can seriously affect the people afflicted and can manifest at any age. Each disease is in isolation rare, but are expressive when different diseases are accounted for. With an early diagnosis, frequently treatments are effective, not only helping patients to have a better life, but also lowering treatment costs of these patients throughout their lives.

In our work with the UFMG laboratory we plan to model several of these diseases. Currently we have modelled adrenoleukodistrofy (ALD). ALD is a disorder of peroxisomal fatty acid beta oxidation which results in the accumulation of very-long chain fatty acids in tissues throughout the body. The most severely affected tissues are the myelin in the central nervous system, the adrenal cortex and the Leydig cells in the testicles [21,22]. Early diagnosis and treatment can reduce or eliminate symptoms during the lifetime of patients, and is essential, but also difficult, due to the fact that the disease is rare and complex to diagnose.

**Figure 4:**
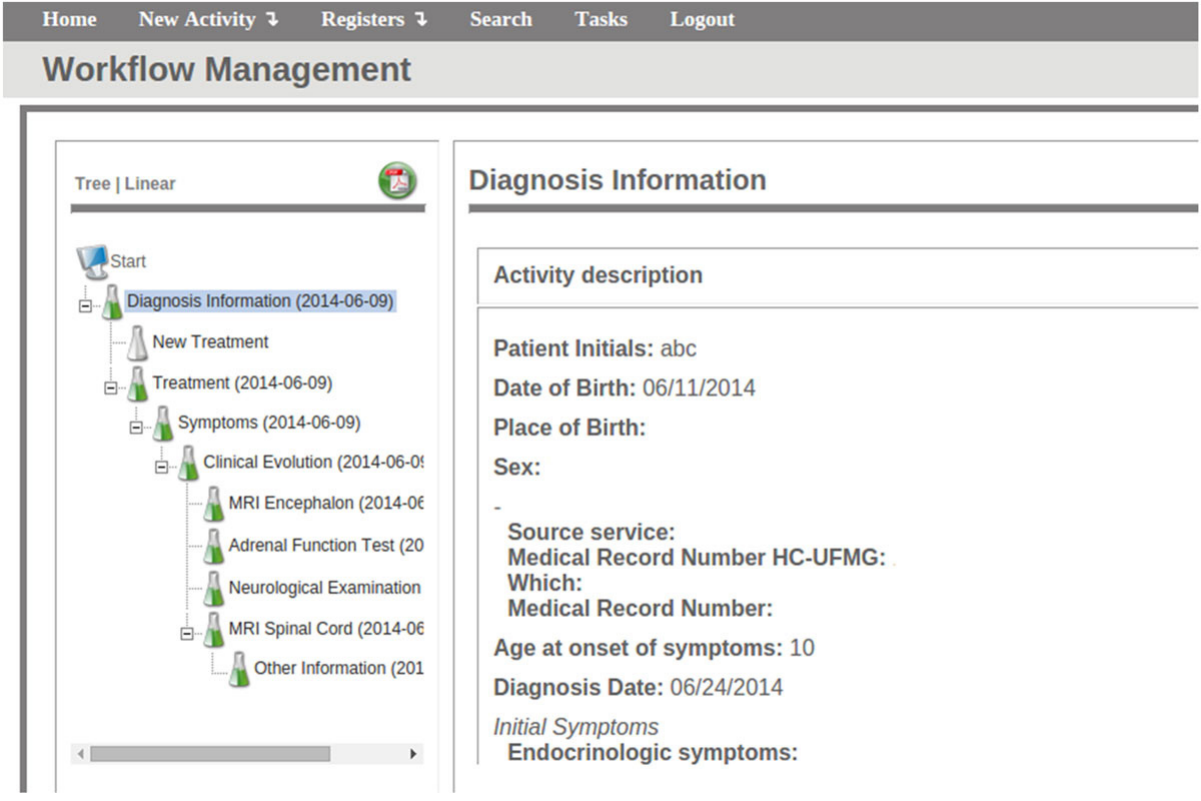
**FluxMed web interface -- Electronic forms for the ALD EHR system**. http://syrah.luar.dcc.ufmg.br/FluxMED -- guest/gu3st

We have modelled the ALD protocol from the Inborn Errors of Metabolism Laboratory and created an EHR system to manage this data (Figure 4).

**Figure 5:**
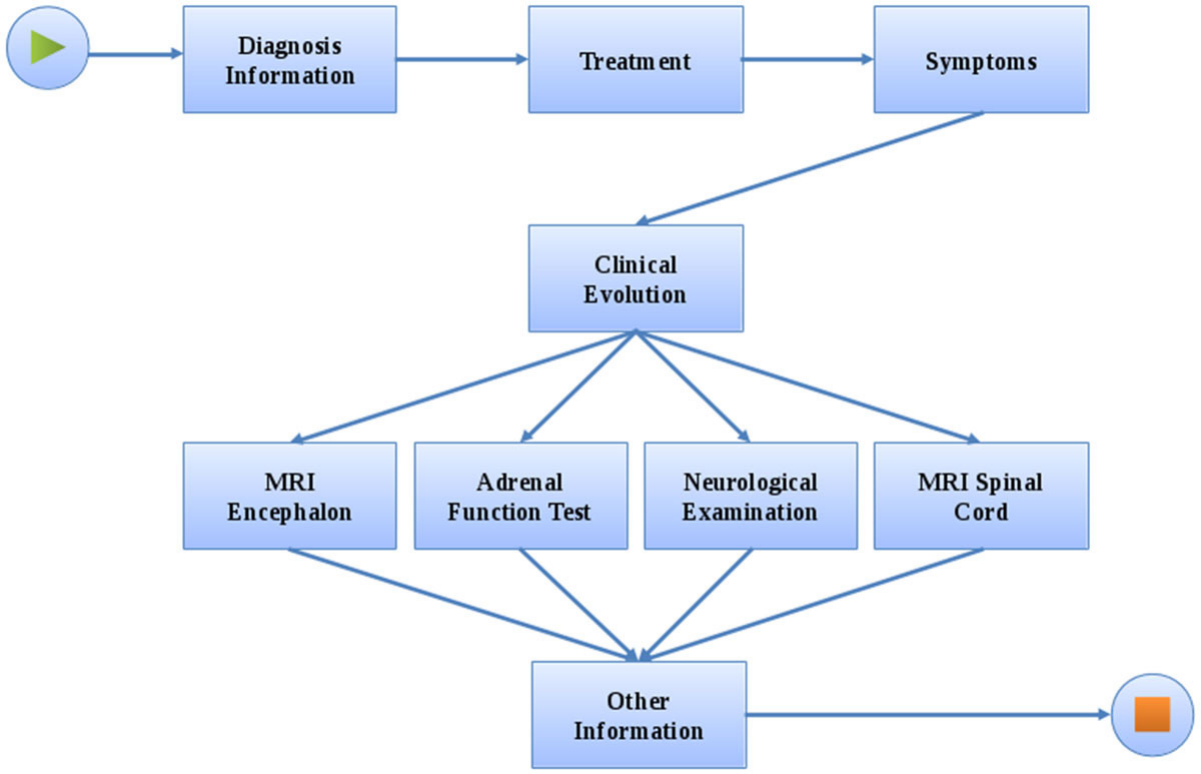
**The Adrenoleukodistrofy (ALD) workflow definition**.

The ALD protocol has 9 activities (Figure 5). The first activity (*Diagnosis Information*) registers both patient identification (patient initials, birth date, place of birth, previous medical record numbers, genotype and other patient important data) and the first diagnosis information, including the first symptoms and exams information. The following step in the system is the input of data about the chosen treatment. In this step the system provides common treatment to the adrenoleukodistrofy in a pre-built list, as well allows the user input his/hers own treatment. This new treatment can also become a pre-built treatment if necessary. *Symptoms *have the same functionality as *Treatment *: the system provides some default symptoms (e.g.: *Fatigue*, *Leg Pain*) and allows the user to add other options. Also, all the required data about the patient *Clinical Evolution *are stored by the system at each visit, like *MRI Encephalon*, results of each *Adrenal Function Test*, the *Neurological Examination *data and Magnetic Resonance Imaging (MRI) of the Spine (*MRI Spinal Cord *form). The system provides, at the end of each of these assays, a field to make general annotations (*Other Information*).

We are currently registering in the system data from patients from the laboratory at UFMG, which number to about 400 per year.

## Discussion

The use of a workflow to develop an adaptable LIMS such as the SIGLa System has resulted in the construction of a very flexible tool. All changes on the activities flow affect only the workflow, since it belongs to an independent layer on the system. Therefore changes to adapt the system to the needs of a new laboratory or facility are easy to implement and do not require any programming skills from the user.

The construction of the NMO, PCM and ALD data management systems represent an important contribution to help improve data collection from patients and can assist significantly in their treatments. FluxMED has a set of important features like an audit and backup system, integration with equipments and report generation, that can assist in medical diagnosis by grouping all information in a tool with easy access and a friendly interface. All data regarding a patient can be easily retrieved from the database using the system increasing precision and efficiency to the analysis of the data.

All of these workflows are highly complex and specialized, building an EHR system for each would not have been practical and would not have been done without the FluxMED system. Each has some characteristics that make it particularly difficult to model using generic content management tools. For example, the NMO system has a complex set of symptoms that have to be registered in the right order to identify the occurrence of the disease. Not only that, but the number of occurrences of each symptom is unbounded. The PCM system has a very large number of exams and questions, modelled as checkboxes. Some are yes/no questions, others require an external table of values, while others require the input of numbers representing the results of exams. The ALD system is only the tip of the iceberg of a EHR system for genetic diseases, and it is being prepared to use a feature called *diagnosis suggestion*, where results from a previous activity are used to suggest the user which next activities should be executed. This feature is essential in guiding the doctor through a number of genetic diseases to identify the correct one. This is ongoing work.

## Conclusions

The advances on medical research and procedures resulted in the generation of a large amount of medical data. The storage and management of this information has become incompatible with the use of paper medical charts and computer flat files, requiring an automated system. The adoption of more powerful tools to address this issue is an immediate need in many clinics and medical offices, particularly for those working on complex diseases that are difficult to diagnose. In this work, we have developed FluxMED, a workflow based system for that purpose with a modern and user friendly interface designed to assist medical doctors on the diagnosis and monitoring of their patients. The system is flexible and can be modified by the user to model different types of diseases, contributing to make diagnosis faster and more reliable, helping to distinguish complex diseases from other related illnesses, shortening treatment times and improving patients quality of life. FluxMED is currently being used in three different laboratories or clinics at UFMG, assisting doctors and patients.

Future work includes an integrated data analysis module that can automatically analyze the data in search of patterns that can assist in the diagnostic and treatment. Currently this analysis is manual. Once this analysis is available a diagnostic suggestion module can be implemented that can compare symptoms of different diseases and suggest a path to follow in the consultation and diagnostic. Currently data from different diseases are not compared.

## Competing interests

The authors declare that they have no competing interests.

## Authors' contributions

ACFC and LAH, constructed and tested the workflow XPDL files. PHB, VG and SVAC developed and implemented the LIMS Flux. All authors have helped in preparing the manuscript.

## Declarations

Publication charges for this article have been funded by FAPEMIG.

All medical data entered in FluxMed has access restricted to the doctors that provided it, unless explicitly stated otherwise. All medical data mentioned in this paper and accessible at the FluxMed web site is fictitious and do not represent actual patients or doctors.

This article has been published as part of *BMC Bioinformatics *Volume 16 Supplement 19, 2015: Brazilian Symposium on Bioinformatics 2014. The full contents of the supplement are available online at http://www.biomedcentral.com/bmcbioinformatics/supplements/16/S19
